# Addressing post-COVID-19 musculoskeletal symptoms through telemedicine: A study protocol

**DOI:** 10.12688/f1000research.122843.2

**Published:** 2023-06-01

**Authors:** Eleuterio A. Sánchez Romero, Josué Fernández Carnero, José Luis Alonso Pérez, Lidia Martínez Rolando, Jorge Hugo Villafañe

**Affiliations:** 1 Department of Physiotherapy, Faculty of Health Sciences, Universidad Europea de Canarias, La Orotava, Tenerife, Canary Islands, 38300, Spain; 2 Department of Physiotherapy, Faculty of Biomedical and Health Sciences, Musculoskeletal Pain and Motor Control Research Group, Madrid, 28670, Spain; 3 Musculoskeletal Pain and Motor Control Research Group, Faculty of Health Sciences, Universidad Europea de Madrid, Madrid, 28670, Spain; 4 Musculoskeletal Pain and Motor Control Research Group, Faculty of Health Sciences, Universidad Europea de Canarias, La Oratova, Tenerife, Canary Islands, 38300, Spain; 5, Department of Physical Therapy, Occupational Therapy, Rehabilitation, and Physical Medicine, Rey Juan Carlos University, Madrid, 28032, Spain; 6 Multidisciplinary Pain Treatment Center ONELIFE, Madrid, Spain; 7 IRCCS Fondazione Don Carlo Gnocchi, Milan, 20148, Italy

**Keywords:** COVID-19; Pain; SARS-CoV-2; Musculoskeletal Disease; Telemedicine

## Abstract

**Objective:** The purpose of the study will be to evaluate the effect of a rehabilitation program on the improvement of patients with post-COVID-19 musculoskeletal symptoms, as well as to quantify the impact of telemedicine that evaluates the evolution of pain, functionality, and quality of life. **Methods:** We will carry out a case-control study in post-COVID-19 musculoskeletal symptoms patients who will undergo a multicomponent rehabilitation program, together with an intervention and a follow-up using programmed telemedicine sessions. Data will be collected on the improvement of functional capacity and quality of life, in addition to assessing the evolution of musculoskeletal symptomatology, as well as pain and psychological variables. The telemedicine sessions will improve user adherence and follow-up, and the results are expected to be disseminated to the scientific community during and after the end of the study.

## Introduction

COVID-19 infection causes various clinical manifestations in patients, including neurological manifestations, ranging from headache, dizziness, neuralgia, and neuropathy, to musculoskeletal symptoms and myalgia. ^1^ Some of the most commonly reported symptoms of COVID-19 include fatigue, cough, fever, ageusia (loss of taste), anosmia (loss of smell), and dyspnea (shortness of breath). ^2^ In addition to these symptoms, some people may experience a headache, chest pain, or palpitations. ^3^ Musculoskeletal alterations cause pain symptoms in COVID-19 patients, appearing to be similar in all countries. ^4^ Individuals with post-COVID-19 pain experience widespread pain due to altered nociceptive processing. ^5^ Prolonged immobilization and mechanical ventilation (MV), as well as the restoration of respiratory and physical functions, may delay the patient’s discharge from the intensive care unit (ICU), or only achieve a partial recovery, resulting in decreased quality of life. ^6^ ICU-acquired weakness (ICUW) impairs the peripheral skeletal and respiratory muscles of critically ill patients. This is one of the most serious consequences of long-term immobilization, resulting in delayed weaning from MV and prolonged hospital stay. ^7^ It has been described that patients hospitalized for COVID-19 infection presented with mild to moderate generalized pain that resembled the pattern of musculoskeletal pain (myalgias or COVID-19-induced muscle pain). ^8^ Therefore, understanding the presence and origin of possible sequelae experienced by post-COVID-19 patients should be an emerging priority for researchers and clinicians. ^9^ Based on these underlying mechanisms of COVID-19 infection, it is very plausible that one of the possible post-COVID-19 outcomes is the development of chronic pain. ^10^ Chronic pain represents another pandemic crisis in modern society due to its high burden and high prevalence within the general population. ^11^ Few data are available on post-COVID-19 sequelae related to the development of pain and potential musculoskeletal repercussions, in contrast to research highlighting other dimensions of health. ^12^ In this context, rehabilitation should be initiated immediately after the acute phase to avoid the progression of hospital-acquired weakness and to achieve rapid functional recovery. ^13^ The pathogenesis of widespread musculoskeletal pain in COVID-19 survivors remains unclear and possibly involves the peripheral and central nervous systems. ^14^

Addressing these sequelae, early exercise and rehabilitation protocols applied during the patient’s hospitalization and after discharge from the hospital can help improve musculoskeletal pain symptoms and prevent functional deterioration. ^15^ Physical activity with multicomponent programs has been shown to have a positive effect on function and weakness in COVID-19 infected patients, in addition to producing improvements in pain. ^16^^,^^17^ COVID-19 has a clear functional impairment among other comorbidities. ^18^

The use of telemedicine improves physiotherapy care by assessing musculoskeletal disorders, ^19^ as well as allowing better dissemination of knowledge by improving access for users who cannot frequently attend their face-to-face sessions or to reinforce therapeutic adherence. ^20^ It facilitates an active role of users, based on personalized risk assessment (biopsychosocial factors), and allows users to be tracked, obtaining data. ^8^

The use of Big Data in health tools opens a great opportunity to move toward monitoring platforms that can offer a more complete, adapted, and updated interaction with the user, under the basis of “more users, more data, and then better feedback that allows personalized care”. Likewise, telemedicine makes it possible to improve the information available on health and self-care. ^21^^,^^22^ The interactive environment aims to create a friendly treatment and learning environment, in addition to improving patient adherence and compliance, as this is directly related to treatment efficacy and preventive actions.

Therefore, the hypothesis will be that post-COVID-19 patients with musculoskeletal symptoms undergoing a rehabilitation program plus telemedicine results in decreased pain and improves functionality and quality of life.

We will also determine the increase in adherence to treatment through the application of telemedicine in post-COVID-19 patients with musculoskeletal symptoms.

## Protocol

### Study design

A case-control study will be carried out between June 2022 and February 2023 with male and female patients impacted by post-COVID-19 musculoskeletal symptoms who will undergo a multicomponent rehabilitation program, together with an intervention and a follow-up using programmed telemedicine sessions. Procedures will be conducted following the Strengthening the Reporting of Observational Studies in Epidemiology (STROBE) statement and checklist. ^23^ The study protocol has been approved by the Ethical Committee of the European University of Madrid (reference number CIPI/21/046). Written informed consent will be obtained from all participants and all procedures were conducted according to the Declaration of Helsinki.

**Figure 1.**
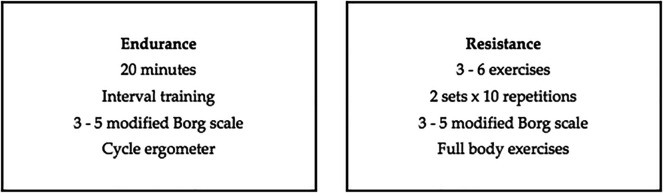
Multicomponent individualized therapeutic exercise intervention planning.

**Figure 2.**
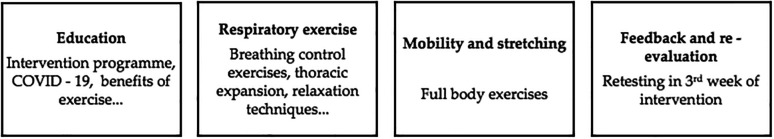
Multicomponent individualized telemedicine intervention planning.

The multicomponent rehabilitation program will be carried out in six weeks of intervention with two weekly face-to-face sessions that will include endurance and resistance exercises ( Figure 1) for both groups (case and control). A once a week telemedicine session will be carried out with the case group only before the face-to-face sessions, consisting of education, respiratory exercise, mobility and stretching, giving them a place to provide feedback and re-evaluate patients mid-treatment ( Figure 2) and will be aimed at assessing improvement and improving therapeutic adherence. The same protocol will be performed once a week in the control group prior to the face-to-face sessions so that comparisons can be made between the two groups. This protocol will be characterized by being progressive and individualized by monitoring the load with validated tools such as the modified Borg scale and Karvonen’s formula. ^24^^,^^25^

### Participants

All participants, whether or not they were previously admitted to ICU by COVID-19, will be contacted by telephone to propose their participation in the study, after which the selected sample meeting the criteria described below that signs the informed consent will be assessed via a comprehensive clinical anamnesis and objective physical examination performed by two expert physical therapists of the rehabilitation department of the Rey Juan Carlos University Hospital of Móstoles, Madrid, Spain, between June 2022 and October 2022. To be included in the study, the patients need to be post-COVID-19 patients (ICU or non-ICU) with musculoskeletal symptoms and be of adult age (over 18 years). Exclusion criteria included: myocardial infarction, uncontrolled arrhythmia, recent pulmonary thromboembolism, terminal illness, patients undergoing lower limb unloading, lower or upper limb fractures in the last three months, severe pain (score greater than 7 on the VAS of 10 points), suffering from the previous pathology that causes neuromuscular weakness, be younger than 18 and older than 65 years old, influenced by medication that does not allow assessment of the real muscular functionality of the patient, patients with cognitive impairment that would prevent them from understanding and collaborating in the performance of the rehabilitation program plus telemedicine, patients with cardiorespiratory instability and uncontrolled arterial hypertension, systemic illness (tumor and rheumatologic diseases), recent unrelated trauma, and limiting psychiatric pathology.

**Figure 3.**
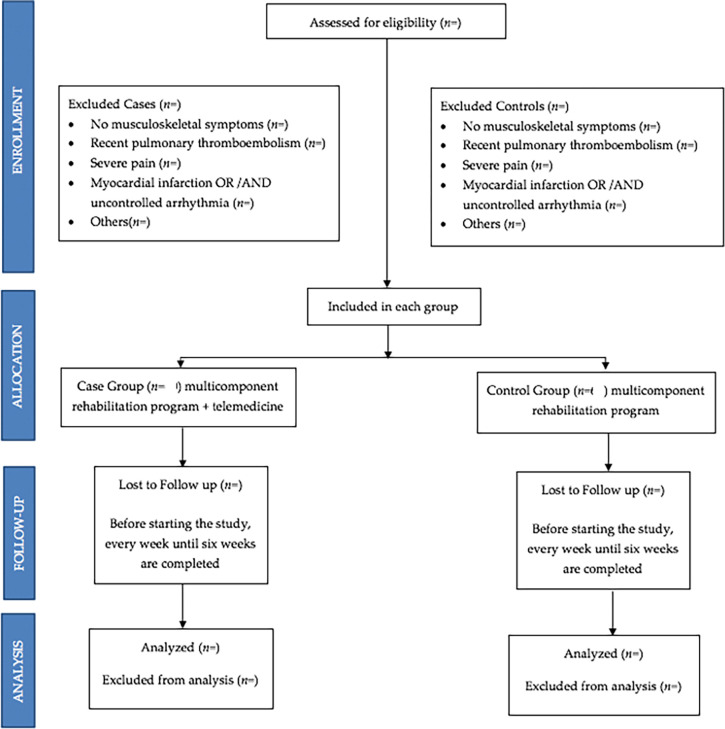
Flowchart of sample selection.

The group of cases and the group of controls will have identical or similar characteristics, except that the cases will be treated with the multicomponent rehabilitation program plus telemedicine, and the control group with the multicomponent rehabilitation program and the same protocol in face-to-face sessions. All subjects will sign an informed consent before inclusion (flowchart, Figure 3).

### Allocation concealment and blinding of researchers

**Figure 4.**
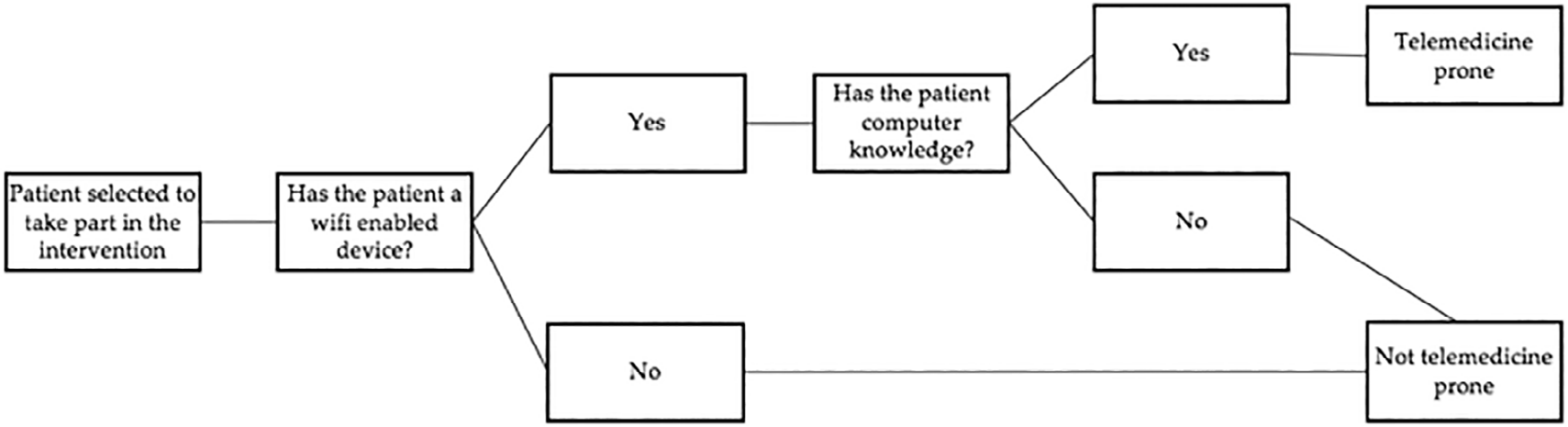
Algorithm to detect samples that are not telemedicine prone.

In this case-control study, 120 patients (of desired homogeneous distribution of males and females) will be included and classified into the following two groups. The initial and final assessment of each potential study participant will be performed by two investigators outside each participant’s intervention group, another investigator will consider the exclusion criteria and follow the algorithm for detecting samples that are not telemedicine-prone ( Figure 4) to stratify the data. An independent researcher with statistical expertise will conduct the analysis of the results obtained.

### Clinical measurements

Patients included in the study will be assessed pre - and post-intervention, using the tools and questionnaires found at https://doi.org/10.17605/OSF.IO/2T3JG in *Table 1* .

*Manual grip strength*

Grip strength will be measured in the affected hand and in the healthy hand (measuring the maximum grip strength). For this measurement, Handgrip strength averaging the result of three attempts with the dominant hand using a Baseline© model pear dynamometer. ^26^

*Quality of life*

Quality of life will be measured with EuroQol-5D-5L ^27^: a test where mobility, self-care, activities of daily living, pain/discomfort and anxiety/depression are assessed.

*Activities of daily living*

Activities of daily living (ADL) will be measured with the Barthel Index, which assesses the level of independence of the subject with respect to the performance of some ADL’s. ^28^

*Assessment of exercise capacity*

Exercise capacity will be measured with a six-minute walking test (6MWT), a sub-maximal exercise test which consists of the patient walking for six minutes along a 30 - meter corridor with two cones marking the distance to be covered while being given a series of cues and monitored for oxygen saturation, heart rate and perceived exertion. ^29^

*Assessment of motor impairment*

Motor impairment will be measured with the Berg Balance Scale (BBS). It determines the ability or inability to safely balance during a series of predetermined tasks. ^30^

*Assessment of perceived pain*

Perceived pain will be measured with the Numerical Pain Rating Scale (NPRS), which measures pain intensity.

*Assessment of neuropathic pain*

Neuropathic pain will be measured with DN4, a questionnaire that assesses the presence of neuropathic pain. ^31^

*Widespread pain*

This will be classified as a continuous numerical variable measured by the Widespread Pain Index (WPI). In this index, the patient must mark with an x the areas in which he/she has presented pain during the last week.

*Psychological variables and self-efficacy*

We will measure psychological variables related to pain sensitivity and other main signs and symptoms, such as kinesiophobia ^32^ and self-efficacy. ^22^^,^^33^^,^^34^ For this purpose, we will use the Chronic Pain Self-Efficacy Questionnaire, in its Spanish-validated version ^35^ and the Tampa Scale of Kinesiophobia, also translated and validated in Spanish. ^36^ Finally, with Beck Depression Inventory (BDI): scale that allows us to measure depressive symptoms and severity of depression in patients older than 13 years. ^37^

### Modulating variables

These variables will be measured by being able to predict a change in the primary measurement results between the first and second measurement of the results or data collection, to facilitate this, the repository found at https://doi.org/10.17605/OSF.IO/2T3JG shows *Table 2* .

‐ Employment status: refers to the subject’s current employment status, and will be classified as a nominal qualitative variable, with the following response modalities: “active”, “unemployed with benefits”, “unemployed without benefits”, “pensioner”.
‐ Levels of physical exercise measures the average amount of physical exercise currently performed per week, and will be classified as a nominal qualitative variable, with the following response modalities: “none”, “less than three times per week”, “three times per week”, “more than three times per week”.
‐ Family economic situation: this variable assesses the average annual economic income of the family unit, and is a nominal qualitative variable, with the following response modalities: “ *more* than 40,000 euros”, between 12,000 euros and 40,000 euros”, “less than 12,000 euros”.
‐ COVID-19’s (SARS-CoV-2) own condition: this variable refers to the current or past presence/absence of illness due to COVID-19 in the subject; it is a nominal qualitative variable, whose response modalities are: “no”, “yes (without symptoms)”, “yes (with symptoms/without admission)”, “yes (with symptoms/admission to ward)”, “yes (with symptoms/admission to ICU)”.
‐ Loss of family members due to COVID-19 (SARS-Cov-2): refers to the loss of family members in subjects due to COVID-19 disease, being classified as a qualitative dichotomous “yes/no” variable.
‐ Chronicity: refers to the number of months that the subjects in the sample have been suffering from symptoms, so it will be classified as a continuous quantitative variable.
‐ Medication: refers to the number of drugs used in the treatment of pain, so it will be classified as a continuous quantitative variable.

### Data analysis

SPSS (RRID:SCR_002865) version 25.0 (IBM SPSS Statistics for Windows; Armonk, NY, USA: IBM Corp) and an α error of 0.05 (95% confidence interval) and a desired power of 80% (β error of 0.2) will be used for statistical analysis. The Shapiro-Wilk test and visual distribution will be used to assess deviations from normality. Parametric analysis will be used in case of normality, given the expected sample size. Then, a comparison of both sociodemographic data and main outcomes between case and control groups will be performed. For case and control groups and for sex, Fisher’s exact test will be used. Pearson’s Chi-square test will compare between case and control groups. In addition, Student’s t-test for independent samples will be used for age and outcomes of the measured variables, and sex and age group. Box plots will be used to illustrate the values of the measured variables of the case and control groups. Univariate correlation analysis will be performed using Pearson’s coefficient (r) to assess the relationship between the variables. Correlations will be interpreted as weak (0.00–0.40), moderate (0.41–0.69) or strong (0.70–1.00). ^38^ In addition, a multivariate predictive analysis will use linear regression and regression trees. Linear regression will be performed using a stepwise selection method and the R2 coefficient to establish quality adjustments. The sample size will be determined by the number of patients admitted to the hospital between June 2022 and February 2023.

## Discussion

Clinical symptoms associated with COVID-19 mainly affect to the respiratory tract, but they manifest heterogeneously from other organ systems including the nervous system. ^4^^,^^39^ We hypothesize that these patients with post-COVID-19 sequelae will demonstrate a pain and potential musculoskeletal repercussions. We expect to find, that the post-COVID-19 sequelae mechanisms might be a feature of this post-COVID-19 population.

This is the first study to use the telemedicine in post-COVID-19 patients with musculoskeletal symptoms. The results of this study can be implemented in clinical practice to help clinicians deal with this challenging patient population. Furthermore, the research will allow the extraction of data on the different patient profiles, symptoms and post-COVID-19 sequelae, in addition to the different risk factors affecting post-COVID-19 patients with musculoskeletal symptoms.

Giacalone *et al.* ^40^ detailed how COVID-19 may invade the central nervous system (CNS) via the viremic spread of SARS-CoV-2, allowing the virus to reach the brain and break through the blood-brain barrier. They also comment on how acute cerebrovascular diseases, such as ischemic and hemorrhagic strokes, have been observed with a higher incidence in patients with severe coagulopathies. Encephalitis, meningitis, seizures, and disseminated acute encephalomyelitis have also been reported in COVID-19 patients. Additionally, peripheral nervous system (PNS) involvement may include myalgia and muscle fatigue, changes in smell, taste, and chemesthetic function, and rare cases of Guillain-Barré syndrome.

In a recent review, Giacalone *et al.* ^41^ detailed how there is evidence to support the use of telemedicine during the COVID-19 pandemic and its potential for permanent integration into healthcare. They assert that telemedicine offers practical and cost-effective advantages, such as breaking down geographic and time barriers, reducing waiting lists, and saving on national healthcare spending.

Patients with chronic pain (20% of the population) have many issues to deal with as there is limited access to specialised pain management centres. Post COVID-19 patients with persistent pain are at risk of not receiving the required recognition and attention by the healthcare system and therefore they will not receive the most optimal pain management for this new pain syndrome. The social repercussions of the current project are imminent since the world should be prepared for a large number (probably millions) of COVID-19 survivors with potential post COVID-19 pain sequelae.

## Conclusions

This project aims to demonstrate that multicomponent approach to musculoskeletal sequelae of COVID-19 will improve pain, functionality and quality of life, ^42^ achieving through telemedicine sessions an improvement in therapeutic adherence and follow-up. The results are expected to be disseminated to the scientific community during and after the end of the study.

## Data availability

### Underlying data

No data are associated with this article.

### Extended data

Extended data for ‘Addressing post-COVID-19 musculoskeletal symptoms through telemedicine: A study protocol’ https://doi.org/10.17605/OSF.IO/2T3JG contains the following data:

- **Table 1.** *Baseline descriptive and clinical variables in the total sample previous intervention*
- **Table 2.** *Baseline predictive variables in the total sample previous intervention*

Data are available under the terms of the Creative Commons Zero “No rights reserved” data waiver (CC0 1.0 Public domain dedication).

## Author contributions

Conceptualization, J.H.V. and E.A.S.R.; methodology, J.H.V., L.M.R. and E.A.S.R.; software, J.H.V.; validation, all authors; formal analysis, J.F.C., E.A.S.R., L.M.R., and J.H.V.; investigation, all authors; resources, J.L.A.P.; writing—original draft preparation, J.H.V, J.F.C., L.M.R. and E.A.S.R.; writing—review and editing, E.A.S.R., J.F.C., L.M.R. and J.H.V.; visualization, E.A.S.R., L.M.R. and J.H.V.; supervision, all authors.; project administration, E.A.S.R. L.M.R. and J.H.V.; funding acquisition, E.A.S.R. and J.H.V. All authors have read and agreed to the published version of the manuscript.
