# Supplementary material for: Addressing post-COVID-19 musculoskeletal symptoms through pulmonary rehabilitation and telemedicine: A study protocol
Source: F1000Res. 2024 Apr 2;11:898. Originally published 2022 Aug 4. [Version 3] doi: 10.12688/f1000research.122843.3 (PMC10958150; doi:10.12688/f1000research.122843.3)
Supplement: Peer review history

# Reviewer report: Reviewer response for version 3

**DOI:** 10.5256/f1000research.164182.r261883

**Published:** 2024-04-30

**Authors:** Ali A. Younis

**Affiliations:** 1 Department of Medicine, College of Medicine, University of Mosul, Mosul, Iraq; 2 University of Mosul, Mosul, Iraq

I appreciate the authors' efforts to address the feedback provided in the previous review. As the revisions are indeed substantial and address the concerns raised earlier, I approve the new version of the article.

Is the study design appropriate for the research question?

Partly

Is the rationale for, and objectives of, the study clearly described?

Yes

Are sufficient details of the methods provided to allow replication by others?

Partly

Are the datasets clearly presented in a useable and accessible format?

Not applicable

Reviewer Expertise:

Rheumatology

I confirm that I have read this submission and believe that I have an appropriate level of expertise to confirm that it is of an acceptable scientific standard.

# Reviewer report: Reviewer response for version 2

**DOI:** 10.5256/f1000research.147632.r251240

**Published:** 2024-03-21

**Authors:** Ali A. Younis

**Affiliations:** 1 Department of Medicine, College of Medicine, University of Mosul, Mosul, Iraq; 2 University of Mosul, Mosul, Iraq

-  The study aims to investigate the effectiveness of a multicomponent rehabilitation program combined with telemedicine in improving pain, functionality, and quality of life in post-COVID-19 patients with musculoskeletal symptoms.
- **Structured methodology:** The protocol outlines a case-control design with clear inclusion and exclusion criteria for participants.  
- **Telemedicine integration:** The study explores the potential of telemedicine for delivering rehabilitation programs and improving adherence in this patient population.
- **Data analysis plan:** The researchers describe a comprehensive statistical analysis plan using appropriate tests based on data types.
- **Potential impact:** The findings could inform clinical practice for managing post-COVID-19 musculoskeletal symptoms and contribute to the integration of telemedicine in rehabilitation.

However, some points need to be considered.

- The title doesn’t refer to rehabilitation which is one of the main objectives of the study.
- The objectives should be mentioned at the end of the introduction.
- In the introduction, It would be better to give more information regarding telemedicine (what is meant by telemedicine, how it is conducted,…).
- The patients included in the study have post-COVID 19 musculoskeletal symptoms, but no clarification is given to what they meant by musculoskeletal symptoms.
- The authors mentioned that “the group of cases and the group of controls will have identical or similar characteristics, except that the cases will be treated with the multicomponent rehabilitation program plus telemedicine, and the control group with the multicomponent rehabilitation” ,   so the group of cases  should have a different study protocol which consists of "education, respiratory exercise, mobility, and stretching, giving them a place to provide feedback and re-evaluate patients mid-treatment.” In this situation, will the group that doesn’t receive   telemedicine   also not have "education, respiratory exercise, mobility, and stretching, giving them a place to provide feedback and re-evaluate patients mid-treatment"? If not, the study groups cannot be compared.
- Blinding participants and researchers might be challenging due to the nature of the intervention (telemedicine sessions).
- While telemedicine use in rehabilitation is growing, the overall approach of combining rehabilitation with telemedicine for post-COVID-19 musculoskeletal problems might not be entirely novel.
-  The six-week intervention period might be insufficient to observe long-term effects on chronic pain and functional limitations.
- The discussion section could be expanded to address potential mechanisms by which telemedicine might improve outcomes.
- The authors might consider including cost-effectiveness analysis alongside clinical outcomes.
- The study plans to recruit from a single center, potentially limiting the generalizability of findings to broader populations.

Is the study design appropriate for the research question?

Partly

Is the rationale for, and objectives of, the study clearly described?

Yes

Are sufficient details of the methods provided to allow replication by others?

Partly

Are the datasets clearly presented in a useable and accessible format?

Not applicable

Reviewer Expertise:

rheumatology

I confirm that I have read this submission and believe that I have an appropriate level of expertise to confirm that it is of an acceptable scientific standard, however I have significant reservations, as outlined above.

# Response

**Published:** 2024-03-23

**Authors:** Eleuterio A. Sánchez Romero

**Affiliations:** European University of Madrid, Spain

*Dear reviewer,*

*Thank you for reading through our manuscript giving us constructive feedback and pointing out important details. The revision process has made the manuscript clearer.*

 1. The title doesn’t refer to rehabilitation which is one of the main objectives of the study.

*Response:Thank you for your comment. The title has been modified as suggested.*

*2. The objectives should be mentioned at the end of the introduction.*

*Response: The sentence corresponding to the objectives of the study has been added at the end of the Discussion section. Thank you.*

*3. In the introduction, It would be better to give more information regarding telemedicine (what is meant by telemedicine, how it is conducted,…).*

*Response: A new paragraph has been added in the Introduction section regarding this, in addition to further detailing the telerehabilitation protocol in the Methods section. Thank you.*

*4. The patients included in the study have post-COVID 19 musculoskeletal symptoms, but no clarification is given to what they meant by musculoskeletal symptoms.*

*Response: The required clarification has been added to the Methods section (inclusion and exclusion criteria). Thank you very much.*

*5. The authors mentioned that “the group of cases and the group of controls will have identical or similar characteristics, except that the cases will be treated with the multicomponent rehabilitation program plus telemedicine, and the control group with the multicomponent rehabilitation” ,   so the group of cases  should have a different study protocol which consists of "education, respiratory exercise, mobility, and stretching, giving them a place to provide feedback and re-evaluate patients mid-treatment.” In this situation, will the group that doesn’t receive   telemedicine   also not have "education, respiratory exercise, mobility, and stretching, giving them a place to provide feedback and re-evaluate patients mid-treatment"? If not, the study groups cannot be compared.*

*Response: Thank you for your comment. Due to your comment, the information in the corresponding section has been modified, clarifying that both groups will receive pulmonary rehabilitation, and in addition, the same telemedicine protocol will be executed in face-to-face mode for the group that is not treated with telemedicine.*

*6. Blinding participants and researchers might be challenging due to the nature of the intervention (telemedicine sessions).*

*Response: Thank you for your comment. In the section "Allocation concealment and blinding of investigators", the single blinding of evaluators is detailed, although it is also detailed that case subjects will not know the treatment of controls, and vice versa.*

*7. While telemedicine use in rehabilitation is growing, the overall approach of combining rehabilitation with telemedicine for post-COVID-19 musculoskeletal problems might not be entirely novel.*

*Response: Thank you for your comment. You are absolutely right in your statement, since it has been some time since the publication of our protocol. However, and although there are only a few weeks left for the submission of the manuscript to a journal, the authors think that the part of having generated post-COVID-19 patient profiles thanks to the use of Big Data, will offer interesting models that will help to better understand the disease.*

*8.  The six-week intervention period might be insufficient to observe long-term effects on chronic pain and functional limitations.*

*Response: Thank you for your comment. It is true what you detail, and although we can no longer modify the protocol because the intervention phase of the study has already ended, the authors will add this point as a limitation in the manuscript submitted to the journal.*

*9. The discussion section could be expanded to address potential mechanisms by which telemedicine might improve outcomes.*

*Response: Thank you for your comment. A paragraph has been added in the discussion regarding this*

*10. The authors might consider including cost-effectiveness analysis alongside clinical outcomes.*

*Response: The authors did not want to add more variables in this study. However, a detailed cost-effectiveness study published by the authors of this study will be presented in the April/June 2024 - Volume 40 - Issue 2 of Topics in Geriatric Rehabilitation journal.*

*11. The study plans to recruit from a single center, potentially limiting the generalizability of findings to broader populations.*

*Response: Thank you for your comment. It is true what you detail, and although we can no longer modify the protocol because the intervention phase of the study has already ended, the authors will add this point as a limitation in the manuscript submitted to the journal.*

# Reviewer report: Reviewer response for version 1

**DOI:** 10.5256/f1000research.134886.r156797

**Published:** 2023-04-18

**Authors:** Marcos Roberto Tovani-Palone

**Affiliations:** 1 Department of Research Analytics, Saveetha Dental College and Hospitals, Saveetha Medical and Educational Trust, Chennai, Tamil Nadu, India

This is an interesting and very well written study protocol on the treatment of post-COVID-19 musculoskeletal symptoms through telemedicine. 

   

 The article is relevant and represents an important intellectual contribution to the literature. The methods used are appropriate and the description of the protocol is complete and quite clear for readers. Furthermore, the proposed objectives are relevant especially for the field of COVID-19. I really appreciate how the authors have described in detail and carefully the design of the protocol as well as all the pertinent methodology. The wisdom and accuracy in the description of data analysis is also another highlight. However, I consider that the discussion could be described in more detail, including new insights. I send below some suggestions that should be especially helpful in generating new ideas and refinement of the manuscript. 

   

 Reference suggestions: 

1. PMID: 34730705 PMCID: PMC8528447 - I recommend this reference given that the article provides an important discussion on the neurological findings of COVID-19. 
2. PMID: 35434056 PMCID: PMC8968610 - I recommend this reference given that the article provides an overview of the importance of ehealth, telehealth, and telemedicine for managing COVID-19 patients.  

However, these two references are only suggestions/examples and the authors should be free to search for other similar articles. 

 Other suggestions: 

- Please describe the full names of all acronyms/abbreviations on their first appearance in the text of the manuscript.  
- Please make it clear when COVID-19 (the disease) or SARS-CoV-2 (the virus) should be used. 
- There are some typographical errors. Please review this. 

Is the study design appropriate for the research question?

Yes

Is the rationale for, and objectives of, the study clearly described?

Yes

Are sufficient details of the methods provided to allow replication by others?

Yes

Are the datasets clearly presented in a useable and accessible format?

Yes

Reviewer Expertise:

Public and Global Health, General Medicine.

I confirm that I have read this submission and believe that I have an appropriate level of expertise to confirm that it is of an acceptable scientific standard.

## References

[1] : Neurological and neuropsychiatric disorders associated with COVID-19. Part I: overview and neurological disorders. *Einstein (Sao Paulo)* .2021;19: 10.31744/einstein_journal/2021CE6448 eCE6448 10.31744/einstein_journal/2021CE6448 34730705 PMC8528447

[2] : eHealth, telehealth, and telemedicine in the management of the COVID-19 pandemic and beyond: Lessons learned and future perspectives. *World J Clin Cases* .2022;10(8) : 10.12998/wjcc.v10.i8.2363 2363-2368 10.12998/wjcc.v10.i8.2363 35434056 PMC8968610

# Response

**Published:** 2023-04-28

**Authors:** Eleuterio A. Sánchez Romero

**Affiliations:** European University of Madrid, Spain

This is an interesting and very well written study protocol on the treatment of post-COVID-19 musculoskeletal symptoms through telemedicine.

 The article is relevant and represents an important intellectual contribution to the literature. The methods used are appropriate and the description of the protocol is complete and quite clear for readers. Furthermore, the proposed objectives are relevant especially for the field of COVID-19. I really appreciate how the authors have described in detail and carefully the design of the protocol as well as all the pertinent methodology. The wisdom and accuracy in the description of data analysis is also another highlight. However, I consider that the discussion could be described in more detail, including new insights. I send below some suggestions that should be especially helpful in generating new ideas and refinement of the manuscript.

 Reference suggestions:

 PMID: 34730705 PMCID: PMC8528447 - I recommend this reference given that the article provides an important discussion on the neurological findings of COVID-19.

 PMID: 35434056 PMCID: PMC8968610 - I recommend this reference given that the article provides an overview of the importance of health, telehealth, and telemedicine for managing COVID-19 patients. 

 However, these two references are only suggestions/examples and the authors should be free to search for other similar articles.

*Response: Thank you for reading through our manuscript. The "Discussion" section has been restructured in accordance to your proposal. The revision process has made the manuscript clearer and heightened the quality.*

 Other suggestions:

 Please describe the full names of all acronyms/abbreviations on their first appearance in the text of the manuscript. 

*Response: Done. Thank you.*

 Please make it clear when COVID-19 (the disease) or SARS-CoV-2 (the virus) should be used.

*Response: Done. Thank you.*

 There are some typographical errors. Please review this.

*Response: Done. Thank you.*

# Reviewer report: Reviewer response for version 1

**DOI:** 10.5256/f1000research.134886.r153318

**Published:** 2022-11-28

**Authors:** Wojciech Glinkowski

**Affiliations:** 1 Center of Excellence "TeleOrto" for Telediagnostics and Treatment of Disorders and Injuries of the Locomotor System, Department of Medical Informatics and Telemedicine, Medical University of Warsaw, Warsaw, Poland; 2 Polish Telemedicine and eHealth Society, Warsaw, Poland

The authors indicate that the experiment program will be a multicomponent rehabilitation program with an intervention and a follow-up using programmed telemedicine sessions.

 A key aspect of telemedicine is the closure of the patient-doctor loop and feedback. The authors of the summary report do not indicate if and what the feedback will be.

 The abstract also states that "results are expected to be disseminated to the scientific community during and after the end of the study." However, the key to presenting the results is to complete the research and obtain the results, hence the question of how the authors will deliver the results without getting them. 

 The authors indicate that  "COVID-19 infection causes various clinical manifestations in patients, including neurological manifestations, ranging from headache, dizziness, neuralgia, and neuropathy, to musculoskeletal symptoms and myalgia." However, the authors do not point to specific symptoms or how to distinguish them from pain syndromes unrelated to COVID-19.

 The authors also propose that the study group should have a different study protocol which consists of "education, respiratory exercise, mobility, and stretching, giving them a place to provide feedback and re-evaluate patients mid-treatment.” In this situation, will the group without distance assessment (I do not write telemedicine because it has not been clearly defined) also not have "education, respiratory exercise, mobility, and stretching, giving them a place to provide feedback and re-evaluate patients mid-treatment"? If not, the study groups cannot be compared.

 The authors report, “Physical therapists involved in face-to-face treatment will not know which sample also has a telemedicine focus." This may suggest an attempt at randomization. So how is randomization going to work? How will patients be allocated to groups? If patients have different symptoms at different locations, how do the authors want to classify them as a homogeneous study group? A checklist?

 The authors also want to compare subjects who are not informed whether they are taking painkillers or other drugs besides telling them whether they are taking medications. Taking painkillers may change the achievement of symptoms planned for collection. The introduction of additional exercises may also change the results significantly, making it impossible to compare the test group with the control group.

 In the reviewer’s opinion, the study protocol requires explicit declarations and ensures the comparability of the test group with the control group.

 Regarding "variables will be measured by being able to predict a change in the primary measurement results between the first and second measurement." What information do the authors expect to gain from the items concerning:

- "Loss of family members due to COVID-19 (SARS-Cov-2): refers to the loss of family members in subjects due to COVID-19 disease, being classified as a qualitative dichotomous “yes/no” variable."
- "Chronicity refers to the number of years that the subjects in the sample have been suffering from symptoms so that it will be classified as a continuous quantitative variable."
- "Medication: refers to the number of drugs used to treat pain so that it will be classified as a continuous quantitative variable."

Is the question about family members supposed to relate to the mental state and mood disorders and the more difficult participation in rehabilitation associated with it? Will the question give information about the duration of the symptoms? Will the question about the number of drugs determine which groups of medicines affecting the study’s results are used by patients?

 A final concern about the submission is how the authors explain the co-author's involvement in the data curation activity in the absence of available data from this study.

Is the study design appropriate for the research question?

Partly

Is the rationale for, and objectives of, the study clearly described?

Partly

Are sufficient details of the methods provided to allow replication by others?

Partly

Are the datasets clearly presented in a useable and accessible format?

Not applicable

Reviewer Expertise:

Orthopedics, traumatology, sports medicine, spinal surgery, telemedicine, teleorthopaedics, telerehabilitation

I confirm that I have read this submission and believe that I have an appropriate level of expertise to confirm that it is of an acceptable scientific standard, however I have significant reservations, as outlined above.

# Response

**Published:** 2023-04-28

**Authors:** Eleuterio A. Sánchez Romero

**Affiliations:** European University of Madrid, Spain

*Response: Thank you for reading our manuscript and for providing constructive comments and pointing out important details. The revision process has made the manuscript clearer and of higher quality.*

 1. The authors indicate that the experiment program will be a multicomponent rehabilitation program with an intervention and a follow-up using programmed telemedicine sessions.

 A key aspect of telemedicine is the closure of the patient-doctor loop and feedback. The authors of the summary report do not indicate if and what the feedback will be.

*Response: We appreciate your comments with the intention of improving the manuscript.* *In "Study design", we add "A once a week telemedicine session will be carried out with the case group only before the face-to-face sessions, consisting of education, respiratory exercise, mobility and stretching, giving them a place to provide feedback and re-evaluate patients mid-treatment ( Figure 2) and will be aimed at assessing improvement and improving therapeutic adherence.”*

*Thank you.*

 2. The abstract also states that "results are expected to be disseminated to the scientific community during and after the end of the study." However, the key to presenting the results is to complete the research and obtain the results, hence the question of how the authors will deliver the results without getting them.

*Response:* *The sentence has been changed to" the results are expected to be disseminated to the scientific community after the end of the study.” Thank you.*

 3. The authors indicate that  "COVID-19 infection causes various clinical manifestations in patients, including neurological manifestations, ranging from headache, dizziness, neuralgia, and neuropathy, to musculoskeletal symptoms and myalgia." However, the authors do not point to specific symptoms or how to distinguish them from pain syndromes unrelated to COVID-19.

*Response:* *Thank you very much for your comment. We have added the following sentence "COVID-19 infection causes various clinical manifestations in patients, including neurological manifestations, ranging from headache, dizziness, neuralgia, and neuropathy, to musculoskeletal symptoms and myalgia. Some of the most commonly reported symptoms of COVID-19 include fatigue, cough, fever, ageusia (loss of taste), anosmia (loss of smell), and dyspnea (shortness of breath). In addition to these symptoms, some people may experience a headache, chest pain, or palpitations.”*

 4. The authors also propose that the study group should have a different study protocol which consists of "education, respiratory exercise, mobility, and stretching, giving them a place to provide feedback and re-evaluate patients mid-treatment.” In this situation, will the group without distance assessment (I do not write telemedicine because it has not been clearly defined) also not have "education, respiratory exercise, mobility, and stretching, giving them a place to provide feedback and re-evaluate patients mid-treatment"? If not, the study groups cannot be compared.

*Response: Thank you very much for your comment. We have added the following sentence "The same protocol will be performed once a week  in the control group prior to the face-to-face sessions so that comparisons can be made between the two groups".*

 5. The authors report, “Physical therapists involved in face-to-face treatment will not know which sample also has a telemedicine focus." This may suggest an attempt at randomization. So how is randomization going to work? How will patients be allocated to groups? If patients have different symptoms at different locations, how do the authors want to classify them as a homogeneous study group? A checklist?.

*Response: Thank you very much for your comment. We have removed that sentence/statement, since we include the same telerehabilitation protocol in face-to-face sessions, we cannot ensure the blinding of the physiotherapists who perform the intervention by the multicomponent rehabilitation program* *.*

 6. The authors also want to compare subjects who are not informed whether they are taking painkillers or other drugs besides telling them whether they are taking medications. Taking painkillers may change the achievement of symptoms planned for collection. The introduction of additional exercises may also change the results significantly, making it impossible to compare the test group with the control group.

*Response: Answer: Thank you very much for your comment. Since the patients belong to the referral hospital, we have the record of their medical history, which contains all their information regarding the consumption of drugs. These will be taken into account, especially those that could influence the results. If the patients meet the inclusion and exclusion criteria, the pertinent statistical analyses will be carried out, since we want to verify whether drugs consumption decreases after the intervention, taking as a reference another study in which we found results in this respect: Sánchez Romero EA, Fernández-Carnero J, Calvo-Lobo C, Ochoa Sáez V, Burgos Caballero V, Pecos-Martín D. Is a Combination of Exercise and Dry Needling Effective for Knee OA? Pain Med. 2020 Feb 1;21(2):349-363.*

*7. In the reviewer’s opinion, the study protocol requires explicit declarations and ensures the comparability of the test group with the control group.*

*Response: Thank you for your comment. We have added the following sentence "The case group and the control group will have identical or similar characteristics, except that the cases will be treated with the multicomponent rehabilitation program plus telemedicine, and the control group with the multicomponent rehabilitation program and the same protocol in face-to-face sessions".*

 8. Regarding "variables will be measured by being able to predict a change in the primary measurement results between the first and second measurement." What information do the authors expect to gain from the items concerning:

 "Loss of family members due to COVID-19 (SARS-Cov-2): refers to the loss of family members in subjects due to COVID-19 disease, being classified as a qualitative dichotomous “yes/no” variable."

*Response: We appreciate your comment.* *In our study, the aforementioned variables are modulating variables. We intend to collect this information and check for a possible influence on the results. Thank you.*

 9. "Chronicity refers to the number of years that the subjects in the sample have been suffering from symptoms so that it will be classified as a continuous quantitative variable."

*Response: We have modified the text to " Chronicity refers to the number of months that the subjects in the sample have been suffering from symptoms so that it will be classified as a continuous quantitative variable.” Thank you*

 10. "Medication: refers to the number of drugs used to treat pain so that it will be classified as a continuous quantitative variable."

 Is the question about family members supposed to relate to the mental state and mood disorders and the more difficult participation in rehabilitation associated with it? Will the question give information about the duration of the symptoms? Will the question about the number of drugs determine which groups of medicines affecting the study’s results are used by patients?

*Response: We welcome your comments. We appreciate your comment.* *In our study, the aforementioned variables are modulating variables. We intend to collect this information and check for a possible influence on the results. We have modified the variables section from "Predictive variables" to "Modulating variables". Thank you.*

 11. A final concern about the submission is how the authors explain the co-author's involvement in the data curation activity in the absence of available data from this study.

*Response: Thank you very much for your comment. We have removed this part from the related section.*
